# The influence of immigrant generation on obesity among Asian Americans in California from 2013 to 2014

**DOI:** 10.1371/journal.pone.0212740

**Published:** 2019-02-22

**Authors:** Shaoqing Gong, Kesheng Wang, Ying Li, Arsham Alamian

**Affiliations:** 1 Institute of Health Administration and Policy, School of Public Policy and Administration, Xi’an Jiaotong University, Xi’an, Shaanxi Province, China; 2 Department of Biostatistics and Epidemiology, College of Public Health, East Tennessee State University, Johnson City, Tennessee, United States of America; 3 Department of Environment Health, College of Public Health, East Tennessee State University, Johnson City, Tennessee, United States of America; CUNY, UNITED STATES

## Abstract

**Objectives:**

We aimed to examine the association between immigrant generation and obesity among Californian adults and Asian Americans.

**Methods:**

We pooled weighted data (n = 2,967) on Asian Americans from the 2013–2014 California Health Interview Survey. Overweight and obesity were defined using body mass indices (BMI) of 25 kg/m^2^ and 30 kg/m^2^, respectively, in non-Asians, compared with BMI of 23 kg/m^2^ (for being overweight) and 27.5 kg/m^2^ (for being obese) in Asians. First-generation or immigrant Asian Americans were defined as those born outside of the U.S. Second-generation Asian Americans were defined as those born in the U.S. with at least one foreign-born parent. All other Asian participants were classified as third-generation or higher. Multiple logistic regression analyses were used with adjustment for age, sex, family income, smoking status, marital status, education, physical activity, and fast food consumption.

**Results:**

Overall, 23.3% of the Asian population was obese, and 40.0% was overweight. The percentage of 1^st^, 2^nd^, and 3^rd^ generation were 72.7%, 22.6%, and 4.6%, respectively. Overall, 1^st^ generation of Asians had lower odds of being obese compared to Whites (OR = 0.34, 95%CI = 0.26–0.45). Multiple logistic regression analyses showed that overall, 2^nd^ generation (OR = 1.69, 95%CI = 1.10–2.60) and 3^rd^ generation (OR = 2.33, 95%CI = 1.29–4.22) Asians had higher odds of being obese compared to 1^st^ generation Asians. Among Chinese, compared to the 1^st^ generation, the 3^rd^ generation had increased likelihood of being obese (OR = 6.29, 95%CI = 2.38–16.6).

**Conclusion:**

Compared to Whites, Hispanics, and Blacks, Asian immigrants are less likely to be obese. Among Asians, 2^nd^ and 3^rd^ generations were more likely to be obese compared to 1^st^ generation. The obesity rate seems to increase the longer Asian immigrants remain in the U.S.

## Introduction

Asian immigrants first came to the United States (U.S.) in significant numbers more than a century and a half ago, mainly as low-skilled male laborers who mined, farmed and built the railroads. They endured generations of officially sanctioned racial prejudice. A first major wave of Asian immigration occurred in the late 19^th^ century, primarily in Hawaii and the West Coast. Since the Immigration and Nationality Act of 1965, Asian American demographics changed rapidly. Over the decades, this modern wave of immigrants from Asia has increasingly become more skilled and educated; a new wave of new immigrants to the U.S. in 2010 were mainly from Asia [1]. In 2014, among a total 42.4 million immigrants in the U.S., Asian Americans accounted for 42.4% [2]. The immigrant population continues to grow. Of all the immigrant populations, Asian Americans were the second largest immigrant group, accounting for 28% of all foreign born populations [3]. In 2011, 79% of Asian Americans aged 18 years and older were foreign-born [4]. Asian Americans are one of the fastest growing populations in the U.S. The Asian population in the U.S. increased by 46% between 2000 and 2010 [5] and will double in population size with a projected increase to more than 43 million people by 2050 [6]; their numbers as a proportion of the U.S. population are projected to grow from 5.8% in 2011 to 9% by 2050 [5].

Immigrants’ health is a function of influences derived from the sending country, the receiving country, and the migration and resettlement experience itself [7]. The existence of a healthy immigrant effect, i.e., that immigrants are on average healthier than the native born, is a widely cited phenomenon across a multitude of literatures including epidemiology and the social sciences [8]. For example, Tu and Colleagues have shown that immigrants to Canada experience lower risk of CVD compared to long-term residents, indicating a healthy immigrant effect, although this protective effect appears to erode slightly over time [7]. Among the few studies that focused on Asians, previous studies that imply the healthy immigrant effect show some evidence that Chinese immigrants have a lower asthma prevalence [9, 10], lower body mass index [10], and overall better general health [11, 12] than native born residents. In the U.S., obesity has become an epidemic over the past two decades [13, 14]. According to the 2013–2014 National Health and Nutrition Examination Survey (NHANES) the age-adjusted prevalence of obesity in the U.S. was 35.0% for men and 40.4% for women [14]. Compared to 2001, obesity prevalence in 2011–2012 has increased among Chinese (3.8% versus 6.1%), Japanese (9.0% versus 15.0%), Filipino (8.8% versus 18.7%), Vietnamese (3.4% versus 6.8%), Southeast Asians (5.8% versus 19.7%), and multiple Asian groups (5.8% versus 12.3%) [15]. Increase in obesity prevalence has also been observed in other ethnic groups (e.g., Whites, Blacks) [16]. However, to date, there is very limited research examining the influence of immigrant generation on obesity among Asian Americans. Identifying factors that have propelled the increase of obesity among Asian Americans is important if we are to effectively combat and prevent obesity nationwide [17].

Research has shown that compared to their native-born cohorts, newly arrived immigrants have better health, but their health declines the longer they remain in the U.S. and become more acculturated [18]. Acculturation is the process by which a minority group adopts the cultural lifestyle and behaviors of the host country [19]. Studies show that some immigrants might possess a health advantage due to migration selectivity and a protective native culture; this protective buffer dissipates by the third generation [20]. Antecol (2006) examined the association between length of residency of immigrants and their obesity. The author found that within 10–15 years after immigration study participants’ BMIs became similar as those of U.S. born; immigrants' BMIs were 2–5% lower than those of U.S. born once they arrived in the U.S. [21].

The aim of this research is to analyze the association of immigration generation and obesity among Asian Americans in California. We analyzed data from a large population-based sample that is representative of Asian ethnic groups: Chinese, Filipino, Vietnamese, Korean, and Japanese [22]. In contrast, most national surveys on health sample a small number of Asian Americans. Further, we pooled weighted data from the latest cycle of California population, i.e., 2013–2014. The results of this study can help to refine the diverse risk profile for obesity among Asian Americans while also contributing to the overall understanding of the impact of migration on chronic health conditions.

## Methods

### Participants

The California Health Interview Survey (CHIS) is a population-based telephone survey designed to produce health data representative of California’s non-institutionalized population. The survey has been conducted biennially since 2001 and continually beginning 2011, and covers comprehensive aspects of health. A detailed technical report of the survey can be found at website http://healthpolicy.ucla.edu/chis/Pages/default.aspx. For CHIS 2013–2014, the landline/list sample household response rate was 14.8 percent; the cell sample household response rate was 16.6 percent. Data on Asian Americans (n = 2,967) of 40,240 whole sample (also including Whites (25,643), Hispanics (n = 7,996), Blacks (n = 1,764)) were obtained from CHIS 2013–2014. CHIS oversampled Asian Americans to increase the precision of estimates for those ethnic groups. Interviews were conducted in five languages [English, Spanish, Chinese (both Mandarin and Cantonese), Vietnamese, and Korean].

We used data collected from Californian adult participants aged 18 to 85 and restricted the sample to Asian descent living in the U.S. and U.S.-born other ethnic groups (i.e., Whites, Hispanics, and Blacks), where they were used as the reference group in our study, respectively. We then excluded the following subjects: 1) Asians who self-identified as being of an ethnicity other than the following large ethnic groups of Asian Americans (Chinese, Filipino, Vietnamese, Korean, and Japanese) or as being of multiple Asian sub-ethnicities; 2) foreign-born Asians who were missing information on the nativity of either parent for studying obesity as outcome variables.

### Study variables

#### Obesity

BMI was calculated using self-reported height and weight. In order to account for racial differences in body fat percentage at the same BMI level, we applied race-specific BMI standards. We used BMI of 25 kg/m^2^ and 30 kg/m^2^ as the cut-off points for the three weight categories (underweight/normal weight (BMI<25 kg/m^2^), overweight (25 kg/m^2^ ≤BMI<30 kg/m^2^), and obese (BMI>30 kg/m^2^) in Whites, compared with BMI of 23 kg/m^2^ (for being overweight) and 27.5 kg/m^2^ (for being obese) in Asians [23].

#### Generational status

First-generation or immigrant Asian Americans were defined as those born outside of the U.S. Second-generation Asian Americans were defined as those born in the U.S. with at least one foreign-born parent. All other Asian participants were classified as third-generation or more [20].

#### Covariates

Age was treated as a continuous variable. Sex and race/ethnicity were treated as categorical variables. Due to small sample size for some subgroups, race/ethnicity included Chinese, Filipino, Vietnamese, Korean, and Japanese. Family income was reported by the adult respondent, usually a parent, and was examined as a percentage of the federal poverty level (FPL), which adjusted for total household income and number of members in the household. Family income was categorized as below 100%, 100% to 299%, or 300% and above of the FPL. Smoking status was defined as current smoker or not current smoker. Marital status was defined as married, never married, or other. Education attainment included three categories: high school, college, and graduate. Physical activity was defined as walking at least 10 minutes for either transportation or leisure over the past 7 days. Fast food consumption was determined by response to the following question: “In the past 7 days, how many times did you eat fast food? Include fast food meals eaten at work, at home, or at fast-food restaurants, carryout or drive through” Response categories included: never, 1–2 times, and ≥ 3 times.

### Statistical analysis

To investigate the characteristics of immigrant generation (e.g., 2^nd^ generation and 3^rd^ generation versus 1^st^ generation) among Asian Americans in California, and the association with obesity, we merged public-use data files of adult samples from the CHIS 2013–2014. All analyses were accounted for complex sampling designs and non-response by employing weights developed by CHIS. Weights are included with the data files and are based on the State of California’s Department of Finance population estimates and projections, adjusted to remove the population living in group quarters (such as nursing homes, prisons, etc.) and thus not eligible to participate in CHIS. When the weights are applied to the data, the results represent California’s residential population during that year for the age group corresponding to the data file in use. Additional information on how to use the CHIS sampling weights, including sample code, is available at: http://healthpolicy.ucla.edu/chis/analyze/Pages/sample-code.aspx.

The descriptive statistics for participant characteristics were reported by Asian ethnicity. Wald Chi-square test was used to examine categorical variables while analysis of variance was used to compare continuous variables between racial/ethnic groups. Potential confounders were controlled if they fulfilled the following three basic properties: 1) be associated with the exposure, 2) be a risk factor for the outcome, and 3) not in the causal pathway of interest. Confounders will also always be associated with the exposure; however, the relationship may or may not be causal, i.e., confounder does not necessarily need to be a common cause of both immigrant generation and obesity in this study [24]. We further fit logistic regression models to obtain adjusted odds ratios (AORs) with 95% confidence interval (CI) of obesity for Asians from different subgroups versus other races (i.e., Whites, Hispanics, and Blacks, as the reference group, respectively). Furthermore, we examined the association between immigrant generation and obesity among Asian Americans using multiple logistic regression analyses that included variables with p value significant at 0.2 in univariate analysis. All tests of significance were two-tailed, with alpha level of 0.05. Analyses were performed using survey data analysis procedures in SAS (version 9.4; SAS Institute Inc., Cary, NC).

## Results

### Characteristics of Asian Americans

Table 1 describes the sample characteristics of Asian adults in California by ethnicity. Overall, 23.3% of the participants were obese, 40.0% were overweight, and 2.4% were underweight. The percentage of 1^st^, 2^nd^, and 3^rd^ generation were 72.7%, 22.6%, and 4.6%, respectively. The majority of the Asian population was aged 18–44 years, had high family income, was not current smoker, was married, had college or graduate degrees, engaged in physical activity, and consumed fast foods during past seven days. Among Asian subgroups, the prevalence of obesity was highest in Filipino (33.8%), followed by Japanese (26.5%), Vietnamese (17.9%), Chinese (17.3%), and Korean (12.8%) (p<0.0001). Koreans had the highest percentage of 1^st^ generation (81.3%), while Japanese had the lowest percentage (27.1%). As for the 2^nd^ generation, the percentage was highest in Japanese (34.8%) and lowest in Koreans (16.1%). As for the 3^rd^ generation, Japanese had the highest percentage (38.1%) while Vietnamese had the lowest percentage (0.2%) (all p<0.05).

**Table 1:** Sample characteristics of Asian adults in California by ethnicity: CHIS 2013–2014 (n = 2,967).

	Overall	Chinese	Korean	Japanese	Filipino	Vietnamese
Weight status, n (%)						
Underweight (0–18.49)	131 (2.4)	47 (3.2)	9 (0.5)	22 (5.2)	8 (2.1)	33 (3.1)
Healthy weight (18.5–22.99)	1371 (34.3)	466 (38.0)	186 (44.2)	179 (36.4)	161 (25.6)	232 (50.3)
Overweight (23.0–27.49)	1530 (40.0)	435 (41.5)	190 (42.5)	193 (31.9)	236 (38.5)	216 (28.8)
Obesity (27.5+)	778 (23.3)	180 (17.3)	60 (12.8)	153 (26.5)	197 (33.8)	73 (17.9)
Immigrant generation of Asians, n (%)						
1^st^ generation	2672 (72.7)	796 (71.7)	387 (81.3)	118 (27.1)	394 (72.3)	509 (79.7)
2^nd^ generation	596 (22.6)	198 (22.0)	33 (16.1)	168 (34.8)	95 (26.0)	39 (20.1)
^rd^ generation	224 (4.6)	49 (6.3)	9 (2.6)	159 (38.1)	12 (1.8)	1 (0.2)
**Sex, n (%)**						
Male	1673 (46.9)	477 (43.8)	197 (43.6)	216 (38.4)	248 (48.3)	249 (48.5)
Female	2137 (53.1)	651 (56.2)	248 (56.4)	331 (61.6)	354 (51.7)	305 (51.5)
**Age**						
18–44 years	1135 (56.2)	350 (60.9)	104 (51.6)	82 (41.5)	220 (52.6)	124 (50.0)
45–64 years	1443 (30.6)	467 (29.0)	131 (27.2)	214 (35.7)	213 (32.1)	233 (36.2)
65 years or above	1232 (13.2)	311 (10.1)	210 (21.2)	251 (22.8)	169 (15.4)	197 (13.8)
**Family income, n (%)**						
<100% FPL	754 (14.6)	204 (13.5)	105 (15.6)	40 (3.9)	83 (13.7)	244 (25.9)
100%-299% FPL	1146 (30.9)	336 (31.4)	177 (42.9)	119 (24.2)	194 (31.0)	184 (32.0)
≧300% FPL	1910 (54.5)	588 (55.1)	163 (41.5)	388 (71.9)	325 (55.3)	126 (42.2)
**Smoking status, n (%)**						
Current smoker	257 (8.8)	55 (9.3)	45 (14.1)	41 (5.2)	51 (9.9)	41 (9.0)
Not current smoker	3553 (91.2)	1073 (90.7)	400 (85.9)	506 (94.8)	551 (90.1)	513 (91.0)
**Marital status, n (%)**						
Married	2233 (54.7)	684 (56.9)	252 (57.2)	288 (52.2)	296 (46.8)	344 (57.2)
Others	815 (12.3)	212 (10.4)	124 (12.7)	158 (12.9)	149 (18.0)	112 (11.3)
Never married	762 (32.9)	232 (32.7)	69 (30.0)	101 (34.9)	157 (35.2)	98 (31.5)
**Education, n (%)**						
High school	1083 (27.2)	315 (30.1)	152 (33.9)	100 (24.5)	111 (18.0)	314 (44.1)
College	1939 (53.1)	522 (46.4)	223 (47.9)	324 (58.5)	426 (70.5)	200 (42.8)
Graduate	788 (19.7)	291 (23.5)	70 (18.2)	123 (17.1)	65 (11.5)	40 (13.2)
**Physical activity**,^**a**^ **n (%)**						
Yes	3058 (79.3)	910 (78.4)	360 (86.8)	411 (80.1)	488 (82.3)	444 (77.8)
No	752 (18.7)	218 (21.6)	85 (13.2)	136 (19.9)	114 (17.7)	110 (22.2)
**Fast food**, **n (%)**						
Never	1982 (43.8)	617 (48.5)	228 (39.9)	245 (41.7)	226 (35.6)	399 (55.6)
1–2 times	1340 (37.2)	389 (35.3)	165 (48.2)	220 (38.5)	251 (36.2)	115 (29.7)
≧3 times	488 (19.0)	122 (16.2)	52 (11.9)	82 (19.7)	125 (28.3)	40 (14.7)

Abbreviation: FPL = federal poverty level.

### Multiple logistic regression analyses for the association between immigration generations and obesity among Californian adults

Table 2 shows results from multiple logistic regression analyses for the association between immigration generations and obesity among Californian adults (Asian Americans vs. Whites, Hispanics, and Blacks, respectively). Compared to Whites, overall, 1^st^ generation of Asians had lower odds of being obese (OR = 0.34, 95% CI = 0.26–0.45). Due to small sample size, Asians were divided into Chinese and other Asian groups. Among Chinese, compared to Whites, 1^st^ generation (OR = 0.26, 95% CI = 0.14–0.46) and 2^nd^ generation (0.24, 95% CI = 0.09–0.64) had reduced likelihood of being obese, but such association disappeared for 3^rd^ generation (OR = 1.08, 95% CI = 0.33–3.49). Among other Asian groups, compared to Whites, being of 1^st^ generation (OR = 0.36, 95% CI = 0.25–0.53) was inversely related to obesity, but such association disappeared for 2^nd^ generation (OR = 0.74, 95% CI = 0.38–1.47) and 3^rd^ generation (OR = 0.78, 95% CI = 0.37–1.63).

**Table 2:** Multiple logistic regression analyses for the association between immigration generations and obesity among Californian adults, CHIS 2013–2014.

	All Asians AOR (95% CI)	Chinese AOR (95% CI)	Other Asians AOR (95% CI)
Asians vs. Whites*			
1^st^ generation	0.34 (0.26–0.45)	0.26 (0.14–0.46)	0.36 (0.25–0.53)
2^nd^ generation	0.59 (0.34–1.04)	0.24 (0.09–0.64)	0.74 (0.38–1.47)
3^rd^ generation	0.86 (0.45–1.64)	1.08 (0.33–3.49)	0.78 (0.37–1.63)
Asians vs. Blacks*			
1^st^ generation	0.16 (0.12–0.22)	0.11 (0.06–0.21)	0.17 (0.11–0.26)
2^nd^ generation	0.26 (0.14–0.46)	0.11(0.04–0.29)	0.32 (0.16–0.66)
3^rd^ generation	0.41 (0.22–0.78)	0.46 (0.17–1.28)	0.39 (0.19–0.81)
Asians vs. Hispanics*			
1^st^ generation	0.24 (0.18–0.32)	0.16 (0.09–0.28)	0.26 (0.18–0.38)
2^nd^ generation	0.46 (0.26–0.81)	0.20 (0.08–0.52)	0.55 (0.28–1.09)
3^rd^ generation	0.68 (0.35–1.29)	0.78 (0.29–2.11)	0.62 (0.29–1.34)

Abbreviations: AOR, adjusted odds ratio; CI, confidence interval.

*The multiple regression analyses were used to adjust for age, sex, family income, smoking status, marital status, education, physical activity, and fast food consumption.

Compared to Hispanics, overall, 1^st^ generation (OR = 0.24, 95% CI = 0.18–0.32) and 2^nd^ generation (OR = 0.46, 95% CI = 0.26–0.81) of Asians had lower odds of being obese. Among Chinese, compared to Hispanics, 1^st^ generation (OR = 0.16, 95% CI = 0.09–0.28) and 2^nd^ generation (0.20, 95% CI = 0.08–0.52) had reduced likelihood of being obese, but such association disappeared for 3^rd^ generation (OR = 0.78, 95% CI = 0.29–2.11). Among other Asian groups, compared to Hispanics, being of 1^st^ generation (OR = 0.26, 95% CI = 0.18–0.38) was associated with decreased odds of being obese, but such association disappeared for 2^nd^ generation (OR = 0.55, 95% CI = 0.28–1.09) and 3^rd^ generation (OR = 0.62, 95% CI = 0.29–1.34).

Compared to Blacks, overall, 1^st^ generation (OR = 0.16, 95% CI = 0.12–0.22), 2^nd^ generation (OR = 0.26, 95% CI = 0.14–0.46), and 3^rd^ generation (OR = 0.41, 95% CI = 0.22–0.78) of Asians had lower odds of being obese. Among Chinese, compared to Blacks, 1^st^ generation (OR = 0.11, 95% CI = 0.06–0.21) and 2^nd^ generation (OR = 0.11, 95% CI = 0.04–0.29) had reduced likelihood of being obese, but such association disappeared for 3^rd^ generation (OR = 0.46, 95% CI = 0.17–1.28). Among other Asian groups, compared to Blacks, 1^st^ generation (OR = 0.17, 95% CI = 0.11–0.26), 2^nd^ generation (OR = 0.32, 95% CI = 0.16–0.66) and 3^rd^ generation (OR = 0.39, 95% CI = 0.19–0.81) were associated with decreased odds of being obese.

### Multiple logistic regression analyses for the association between immigration generations and obesity among Asian adults

Table 3 shows results from multiple logistic regression analyses for the association between immigration generations and obesity among Asian adults. Overall, 2^nd^ generation (OR = 1.69, 95% CI = 1.10–2.60) and 3^rd^ generation (OR = 2.33, 95% CI = 1.29–4.22) were associated with higher odds of being obese compared to 1^st^ generation. Among Chinese, compared to 1^st^ generation, 3^rd^ generation had increased likelihood of being obese (OR = 6.29, 95% CI = 2.38–16.6). Among other Asian groups, compared to 1^st^ generation, 2^nd^ generation were associated with increased likelihood of being obese (OR = 1.99, 95% CI = 1.14–3.47).

**Table 3:** Multiple logistic regression analyses for the association between immigrant generation and obesity among Asian Americans, CHIS 2013–2014.

	All Asians AOR (95% CI)	Chinese AOR (95% CI)	Other Asians AOR (95% CI)
Immigrant generation*			
1^st^ generation (ref)			
2^nd^ generation	1.69 (1.10–2.60)	1.47 (0.57–3.80)	1.99 (1.14–3.47)
3^rd^ generation	2.33 (1.29–4.22)	6.29 (2.38–16.6)	1.71 (0.85–3.44)

Abbreviations: AOR, adjusted odds ratio; CI, confidence interval.

*The multiple regression analyses were used to adjust for age, sex, family income, smoking status, marital status, education, physical activity, and fast food consumption.

## Discussion

This study is among the very few studies to examine the association between immigrant generation and obesity among Asian Americans. We found that, using WHO Asian cut points for obesity, overall, 23.3% of the Asians were obese, and 40.0% were overweight. The percentage of 1^st^, 2^nd^, and 3^rd^ generation in the study were 72.7%, 22.6%, and 4.6%, respectively. Compared to other races (i.e., Whites, Hispanics, and Blacks), Asians were less likely to be obese, especially for 1^st^ generation of Asian Americans. Although 2^nd^ and 3^rd^ generations of Asians were also associated with reduced obesity prevalence as compared to other races, the magnitude of the association decreased compared to the 1^st^ generation of Asians. For example, 1^st^ generation, 2^nd^ generation, and 3^rd^ generation of Asians had lower odds of being obese compared to Blacks. This finding is further supported by the observation that among Asian adults, 2^nd^ generation and 3^rd^ generation were associated with higher odds of being obese compared to 1^st^ generation. These findings are consistent with previous studies, e.g., compared to their native-born cohorts, newly arrived immigrants’ health declines the longer they remain in the U.S. and become more acculturated [17]. All the evidence suggests an increased risk of being obese among immigrants due to lifestyle changes closer to the U.S. born population with increasing generation, i.e., indicating that acculturation to U.S. lifestyles contributes to their heightened obesity risk.

Asian Americans have been reported to be less obese than other major U.S. ethnic population groups [25]; the latter may be due to the high proportion of immigrants in the Asian American population. Studies on obesity in Asian Americans have found that compared to U.S.-born Asian Americans, foreign-born Asian Americans are significantly less overweight and obese, though increased duration of residence in the U.S. among foreign-born Asian Americans is correlated with increased obesity [26–28]. Studies of Asian Americans by immigrant generation have observed a trend of increasing obesity with later generations [25, 29]. Although it is assumed that U.S. environmental characteristics and acculturation to western lifestyles (e.g., diet and exercise) may explain the phenomenon, very limited studies have been conducted to link immigrant generation and subsequent obesity in Asian American adults. The similarities and differences with findings of previous studies on migrant status and obesity may be due to study designs (e.g., cross-sectional, cohort studies), sample size for the study population, and measures of study variables (e.g., definition of migrants status, cut-off points used for defining obesity among Asians).

Although immigrants likely arrive in the U.S. with adverse social and economic profile, they have health advantage, which may reflect migration selectivity [27, 30, 31] or the protective culture of immigrants. This encourages healthy behaviors and strong social support systems [32, 33]. Nevertheless, immigrants and their subsequent generations lose at least some of this initial health advantage [21, 34]. With regard to obesity, the differences in its prevalence and related risk factors have been reported between immigrants and U.S. born population [29], leading to different obesity prevalence between Asians and other races.

Immigration selection, Asian culture (diet and life style), and a tight social network among first- generation families may have a protective role in health outcome, e.g., they may protect male immigrants from an increased risk of type 2 diabetes in the U.S. [20]. BMI is, in particular, lower in many sending countries than in North America or Western Europe [25]. This study is mainly focused on Asians, and compared Asians with other ethnic groups (Whites, Hispanics, and Blacks, as the reference group, respectively). For example, compared to Asians immigrants, White immigrants may be less likely to change significant diet and life style in later generations. Thus, using Whites or other ethnic groups as reference groups that did not exclude their immigrants may not significantly bias the results. Acculturation is considered to likely modify health and behavioral risks of immigrants, leading to decreased odds of being obese from 1^st^ to 3^rd^ generations [29]; this is consistent with a recent study where health advantage in some immigrants due to migration selectivity and a protective native culture seem to dissipate by later generations [20]. The association of immigrant generation and obesity was also observed in Chinese and other Asian groups.

In addition, some studies have suggested a positive association between immigrant generation and other health outcomes. For example, a study found that immigrant generation is associated with increased diabetes risk among adults of Mexican origin. Compared with 1^st^ generation Mexican immigrants, U.S. born second- and third-generation individuals had higher diabetes risk [35].

Immigrants’ advantage in body composition is driven by the selection of healthy people to immigrate to the U.S. and the selection of unhealthy people to return to their home country. Immigrants’ initial body composition advantage, however, is gradually eroded by the exposure to the high risk of obesity in the U.S. [36]. Previous studies have reported that immigrants have a lower BMI than the native born [27, 37], and further document that immigrants who have spent less than 10 years in the U.S. have a significant lower BMI than those with a longer duration of residence [27]. Compared with duration of migration, age at immigration is also important. It is likely that healthy behaviors (e.g., less exposure to smoking, better diets, and more light or moderate intensity physical activity) were culturally ingrained prior to immigration [10]. Immigrants may have developed these habits before immigrating, and likely have retained them if they immigrate at older age and live in the potentially protective ethnic enclave, which could be protective because they facilitate access to services, food, and social opportunities [38]. Roshania et al. (2012) shows that immigrants younger than 20 at arrival in the U.S. may be at higher risk of overweight/obesity with increasing duration of residence than those who arrive at later ages. Obesity prevention among young U.S. immigrants should be a priority [39].

The strengths of this study include the large CHIS sample that used people from many ethnic groups and provides health-related information for most large and small racial and ethnic population in particular Asian subgroups. In addition, CHIS is conducted in different languages to represent diverse population. Some limitations also need our attention. First, our analyses were limited to the CHIS public use file, and thus we did not have access to information such as parents’ national origins or documentation status. Second, CHIS only interviews respondents in residential settings, which do not include homeless individuals and people living in group quarters such as group homes, dormitories, jails, and prisons. Third, the original sample size is small, and due to small sample sizes for some subgroups, Asians group was only dichotomized into Chinese and other Asians as a whole, proving limited information for Japanese, Koreans, Filipino, and Vietnamese. Fourth, our data are subject to self-report bias. Self-reported height and weight used in this study may underestimate the prevalence of obesity [40]. Measured obesity rates were reported to be dramatically higher compared to self-reported estimates [41] and were consistent with overall national obesity prevalence of 34% from NHANES 2007–2008 [42], highlighting the importance of using measured height and weight when determining population estimates. Fifth, the use of a new definition of a confounder may need some attention [43, 44], which does go deeper and beyond the methods employed in the current manuscript. The new definition of a confounder pertain to approaches that include creating a framework of concepts and methods for colliders, instrumental variables, d-separations, backdoor paths and, notably, Directed Acyclic Graphs [43]. This is because the proposed new definition of a confounder is said to 1) better distinguish between mediators and confounders, so that direct and indirect effects are more clear and 2) help limit adjusting for a mediator that generate selection bias, as in the birth weight paradox [43]. Additional benefits of using the new methods and definitions of confounders are described elsewhere [44]. Finally, CHIS is a California-based random-digit dial survey of population health. Low response rates for the years included in this study limit generalizability. Additionally, our results are not generalizable to the U.S. population since CHIS only samples the California population.

## Conclusion

Compared to non-immigrants, immigrants are more likely to have obesity. Among Asians, 2^nd^ and 3^rd^ generals were associated with higher likelihood of being obese compared to 1^st^ generation. The obesity rate seems to increase the longer Asians remain in the U.S. due to being more acculturated.

## Supporting information

S1 File (RAR)
